# Exploring Needs and Expectations of Spouses of Addicted Men in Iran: A Qualitative Study

**DOI:** 10.5539/gjhs.v6n5p132

**Published:** 2014-05-19

**Authors:** Soodabeh Joolaee, Zhila Fereidooni, Naeemeh Seyed Fatemi, Mohammad Hassan Meshkibaf, Jila Mirlashri

**Affiliations:** 1 Centers for Nursing Care Research, Nursing and Midwifery School, Iran University of Medical Sciences, Tehran, Iran; 2 School of Nursing and Midwifery, Iran University of Medical Sciences, Fasa University of Medical Sciences, Fasa, Fars, Iran; 3 Department of Biochemistry, Fasa University of Medical Sciences, Fasa, Fars, Iran; 4 Centers for Nursing Care Research, Nursing and Midwifery School, Tehran University of Medical Sciences, Tehran, Iran

**Keywords:** addiction, expectation, need, spouse

## Abstract

Addiction is one of the majore problems that affect everyone in the society especially the spouses of addicted men who have to face a large number of problems which are the consequences of their husband’s addiction. This qualitative study was conducted to explore the needs and expectations of women who are living with their addicted husband in Iran. Twenty-four spouses of addicted men participated in this study. The participants were interviewed and each interview was analyzed via the content analysis method. The results of this study showed that the women’s difficulties were related to their approach to the treatment, or their husbands’ response to the treatment, financial constraints and emotional and informational needs. Moreover, these Iranian women expected more stringent control by the government on the phenomenon of addiction and drug trafficking with a view of having a drug-free country. The needs and expectations of the wives of addicted men are context-based and should be assessed separately between individuals, families, and communities. In addition to the addicted person, it is vitally important that the health of the family members of drug addicts be taken into account and for whom supportive services be provided.

## 1. Introduction

Addiction is the continued use of mood or behavior-altering substances despite its adverse dependency consequences (Angres & Angres, 2008), or a neurological impairment leading to such behaviors (American Society for Addiction Medicine, 2012). Drug abuse is associated with such co-existing problems as mental health, isolation, deprivation, and unemployment. Substance abuse can have a negative impact on the family, social interactions, and communities.

Addiction is a disease that affects both mind and behavior. Researchers have identified a lot of biological, psychological, and environmental factors that contribute to the development and progression of addiction. Scientists have drawn their attention upon this issue to develop a variety of effectively preventive, therapeutic, and recovery supportive approaches to reduce drug abuse. Expansion of addiction research and application of those results to improve care are the keys to successfully ameliorating those problems (National Institute on Drug Abuse, 2008).

The presence of a drug addict in the family is an extremely stressful experience which can affect the economic condition, physical health, psychological well-being, social life, and relationship of the whole family. These factors often mean that families need help in their own right to be able to cope with what is a usually ongoing, long-term issue (National Treatment Agency for Substance Misuse, 2008). It ought to be both surprising and shocking that there has been so little in the way of a co-ordinated response to families living with the drug misuser’s (Barnard, 2007).

The impact of drug abuse creates a range of needs which can vary among families and their members as well (Macdonald, Russell, Bland, Morrison, & De La Cruz, 2002). There is a paucity of information in Iran on the needs of the family members of drug addicts and the methods of meeting these needs. On a nation scale, our ultimate goal should be to relieve the burden, not only the health burden of substance abuse but also its many concomitant adverse consequences on individuals and society at large. Prospectively, we can change people’s perceptions and replace stigma and shame with a new understanding of addiction as a treatable disease much like any other medical disorders and the one which demands a broad but focused, intense, and sustained public health solution (National Institute on Drug Abuse, 2008).

According to the United Nations’ Office on Drugs and Crime, about 3.3–6.1% of the world’s population aged 15–64 used illicit substances at least once in the previous year (World Drugs Report, 2011). The use of drugs, mainly opium, in the Iranian population dates back to several centuries ago. However, the recreational use of opium is prevalent in many parts of the country even today (Mokri, 2002; Mirlashari, Demirkol, Salsali, Rafiey, & Jahanbani, 2012).

It is hard to accurately estimate how many people may be affected by substance abuse (National Treatment Agency for Substance Misuse, 2008). The records of Iranian addicted population are not accurate since some restrictive rules and cultural tendency do not openly admit the addiction (Mokri, 2002). However, recent official reports confirmed that there are two million drug abusers while unofficial reports have estimated that there are three to four million ones (Tafreshi, 2012). On a conservative estimate, every substance abuser will negatively affect at least two of their close family members (Velleman & Templeton, 2007). This suggests that between four and eight million Iranian family members (the addicts’ wives, children, parents, and siblings) are somehow involved with the negative influences of the drug abusers. This problem has turned into malignant socioeconomic, psychological, and familial phenomena (Tafreshi, 2012).

About 47% of Iranian drug users are married; hence, the spouses and children of drug users are likely to be particularly vulnerable groups affected by the negative consequences of drug abuse. These people should be considered in policy making and research studies (Darrodi, Bahrami, & Bahari, 2010). In Iran, socially and traditionally, women are not much addicted with drug due to some restrictions imposed on them by men, husbands, culture, religion, etc. In a way, they are usually dependent. Accordingly, in this study, the researchers have focused on women suffering the scourge of their addicted husbands. The divorce is unacceptable in Iranian culture; consequently, women are forced to tolerate their addicted husbands with all their problems and difficulties. In this research, most of the participants are unemployed; therefore, they are dependent on their husband’s income and unable to sustain family life by themselves.

Women’s health constitutes the foundation of family and community health more generally, (MacKian, 2008; Parvizy, Naseri, Seyed Fatemi, & Ghasem Zadeh Kakroudi, 2010). Information about the needs and expectations of Iranian women living with addicted men are currently scarce. A good understanding of needs and expectations of these women are essential to help families become more resilient and minimize the addiction consequences.

The early research on difficulties of spouses of addicted men was undertaken by Orford and Edwards, in the 1960 and early 1970s which dealt with their alcoholic husbands. One of the key findings of their study was that ‘marital cohesion’ (incorporating ideas of affection, participation in household tasks, how the wives viewed her husband when he was sober and the degree of optimism held about the future of marriage) was not significant (but dependent) indicator of positive outcome for the husband’s drinking (Velleman, 2003).

Ray, Mertens, and Weisner (2007) compared two groups of similar family members; with substance misuse and no substance misuse. The results of their study showed that the other family members of people with substance misuse more likely to be diagnosed with depression and substance abuse.

Although during the past 3 decades, there has been increased recognition by researchers that families can play key role in substance misuse treatment, in terms of: preventing and/or influencing the course of the substance misuse problem, improving substance-related outcomes for the user and other family members, but implementation of evidence based practice remains low and drug policies do not adequately address the needs of family members or how they can be supported (Copello, Templeton, & Velleman, 2006).

Among sparse Iranian research, a study which found that mistrust, humiliation and feelings of failure, embarrassment and shame, fear and anxiety, insecurity, hopelessness and aggression are more prevalent among women with addicted husbands than that of women with non-addicted husbands (Nouri, 2011). In another study the researchers, compered families of addicted fathers with families of non-addicted fathers. They have found father’s addiction; will affect negatively the socialization of other family members, negatively affect the social relationships of family members, disrupt the family’s economic status. They concluded addicted father rather than having a supportive and economic role in the family turns into a supportable and controllable person (Rahgozar, 2012).

## 2. Method

The present study, which was part of a doctoral dissertation, aimed at understanding the impact of addiction on the spouses living with an addicted husband, their needs and expectations, and their social support. To carry out the research, a purposive sampling technique was used. The participants were 24 wives of addicted men who attended the De- Addiction Clinic (treatment and rehabilitative centers for drug abusers like opiate, its derivatives, chemicals and industrials products eg. Meth Amphetamines), affiliated with Tehran University of Medical Sciences. Having been informed about the objectives of the study, the subjects were told that they could discontinue their cooperation with the researchers at any step of the study whenever they wished. In addition, they were assured that their imparted information would be kept secret when the results would be published. Meanwhile, those who agreed to participate were asked to sign a consent form. The permit for conducting the study was issued by Tehran University of Medical Sciences. Furthermore, the ethical approval was obtained from the Ethics Committee of Tehran University of Medical Sciences. In order to elicit information from the subjects, semi-structured interviews were conducted by using an interview guide. Each interview began with warm-up questions followed by openended ones which assessed the impact of the husband’s drug addiction on the spouse, her needs and expectations and her social support. All interviews were conducted in Persian which was the mother tongue of all the participants. The interviews lasted between 45 and 130 minutes. It took the researchers 22 months to gather the data. The average age of the 24 participants was 38.4 years (range 26 to 57). They had an average of 2.0 children (all but one had children, mostly one or two, although two had six children each), (The other demographic data was shown in Table 1).

**Table 1:** Demographic Information of All 24 Participants (Spouses of Addicted Men)

Educational Level		Spouses Job		Economical Background		Husband Type of Addiction		Husband Type of Job	
Level	NO	Kind of Job	NO	Background	NO	Type	NO	Type	NO
Primary Education	1	Housewife	18	Family Income Lower Than *US$ 200-300 a Month	10	Opium	13	Unemployed	6
Secondary Education	6	Government Employees	4	Family Income of The Equivalent of US$200-300 a Month	13	Opium Plus Shireh or Opium Plus Shireh and Cannabis	2	Unskilled and Unstable Jobs	5
Diploma (Standard 12 Level)	14	Own Business	1	Family Income MoreThan US$ 500 a Month	1	Opium Plus Hreoin	1	Non-Government Employment When Available	6
University Education	3	Retired	1			Opium crack	2	Government or Stable Private Employment	4
						Amphetamine	5	Retired	3
						Amphetamine Plus Opium crack	1		

* 250,000 Rial, is Approximately Equivalent to 1 US Dollar.

The reliability and validity of the study were examined by using the criteria suggested by Guba and Lincoln (Streubert & Carpenter, 2011). The credibility was thought to have been established through the sufficient cooperation and interaction which the authors had with the participants. The principal researcher checked the dependability of analysis through the continual process of consulting the thesis supervisor (JS), associate professors (SN, MMH and MJ), and other experts who reviewed the material and whose additional comments were incorporated. Conformability was aimed for by attempting to set aside presumptions and prejudices. In addition, the validity of the findings was supported by the participants, to all of whom was sent their own transcribed and coded interview for further checking.

## 3. Results

The present study explored the needs and expectations of the wives of addicted men and their difficulties. The results are discussed in two different categories as follows:

### 3.1 Needs

#### 3.1.1 Support for the Treatment of the Addicted Husband

Almost for all the women who had participated in this qualitative study, solving the husband’s addiction problem was considered as a first priority. The issue of treatment and care was important for the participants, but most of them did not know how to deal with the issue. Some of them thought that there was no treatment for addiction. Some were seeking help and support from government officials or any other individuals who could give them a hand. Some of the wives were dependent on their family and relatives though they were not of much help.

Although Iran has made progress in terms of medical care, some of the women believed that drug and medical treatment not only had no effect on their husband’s addictions but also worsened their situation, especially when it comes to the problem of addiction relapse. For the better outcome beyond medication and medical therapy, sometimes the wives and addicts need to undergo psychosocial treatment and education.

The health service charges demanded by the country’s health care system is reasonably low, but many of the women stated that they could not afford the costs. They demanded free counseling and treatment for the drug users and themselves. Some participants in the study suggested that they should be under the coverage of health insurance companies.

Overall, the women believed that a coordinating effort is needed to solve the problem of addiction and its consequences.

*“I need support to solve my husband’s addiction. This is my priority.”* (p2)

#### 3.1.2 Instrumental and Financial Needs

The women also spoke about the financial and economic needs. They stated that it is difficult for them to meet their basic needs of life. It should be noted that the instrumental and financial needs were increased at the time of treatment follow-up. Nevertheless, to preserve their family dignity, based on the cultural and traditional beliefs, some spouses may suffer the burden without making their financial problems known.

*“I do need financial help especially when I come here (Treatment and Rehabilitative Center for addicts). I’ve even reached a point where I sold my child’s pieces of gold. I’ve sold everything (adding in grief), but I’ve not asked financial help”*. (p11)

3.1.2.1 Support to Have a Shelter

Many of the wives interviewed had no shelter of their own. In addition, they had to pay a large percentage of their low income on mortgage or rent. They said that if they did not have housing problems, it would be easier for them to manage other economic problems. On the other hand, some of the wives believed that if their first-degree relatives such as their parents or parents-in-law provided temporary financial support, it would be of great help and ease the financial burden to some extent.

*“The most important thing that I need is a home; a home is a shelter for my children.”* (p5)

3.1.2.2 Support to Have Job Opportunities

The wives were in great trouble to highlight and talk about their addicted husband’s unemployment or job instability. In fact, they needed support to maintain a job. They also mentioned that the main causes of their husband’s relapse after detoxification and increased anti-social behavior were poverty, unemployment, and economic pressure. The women believed that if the government or private sectors failed to provide job opportunities for their adult children, they would be at risk of substance abuse, too.

*“My husband is unemployed. No income at all. At least, we need an opportunity to work and have a source of income.”*(p17)

#### 3.1.3 Emotional Needs

The participants believed that their husbands were living in stupor, while the rest of the family had to be conscious and live in reality. As a result, the addiction of their husbands’ placed a heavier burden on the rest of the family. Shame, shyness, cultural norms, social isolation, and secrecy were all factors that exacerbated the psychological burden on the spouses of addicted men. The women expressed their needs for emotional support, peace and tranquility, so they will be able to cope with the stress caused by their husband’s substance abuse.

*“I was ashamed; no one has ever heard me talking about my husband’s addiction. There should be a place to cry and somebody to speak to; someone to empathize with you; someone to help you relieve your psychological burden. I need emotional support very much.”* (p24)

*“I had to hide my husband addiction problem because of the social stigma. This secrecy has placed a great deal of pressure on me. I need to be loved. I want to be embraced. I need to talk about my husband’s addiction; I need to get this heavy burden off my shoulder.”* (p10)

#### 3.1.4 Informational Needs

A large number of the women said that they needed a training program to improve their knowledge and information about how to deal and live easily with an addicted husband during the crisis. They also needed to learn how to encourage their husbands to seek treatment and speed up their recovery. The women were in need of information related to the recognition of the addiction phenomenon, how to behave towards the addicted husband during treatment, and how to achieve physical and mental balance with the least harm to their husbands and themselves. Some of the participants in the present study stated that they had tried to seek information, guidance, and counseling, but there was little information or guidance available.

*“I went to a de–addiction clinic to ask how I could help my husband when he refused to come to the clinic to start his treatment. They advised me not to say anything to my husband, He would be alright himself. Their advice was not effective at all.”* (p1)

*“I did not understand the addiction phenomenon. I suffered very much. I needed someone to tell me what to do. I needed education, and I needed an insight into the addiction phenomenon.”* (p13)

*““I was sensitive”. Whenever he smelled of opium, I was checking his clothes and pockets to prove he used opium or not. I was wrong; I did not know about the addiction. This led my husband to turn to something else that did not smell but was more dangerous like methamphetamine (glass) and opium crack.”* (p19)

3.1.4.1 Support to Optimize Decision-Making

Many of the women in the present study said that they did not know what to do; they were under a great deal of stress. They had different strategies for coping with their husband’s addiction and its devastating impacts on the other family members. Participants said that although some of their strategies were useful, some others were not functional in urging their husbands to seek treatment, instead they worsened the situation. The participants acknowledged that by taking wrong steps and decisions under the pressure of their husband’s addiction, they might have caused more harm to their husbands, children, and family. To choose the most effective strategies, the women said that they needed professional guidance, counseling, and training programs.

*“It seems I am stuck at a crossroads; I do not know what to do in my life.”* (p8)

*“When drug addiction enters the family, the family life becomes really miserable. We do not know how to end this problem.”*(p5)

*“I was nervous; the psychologist talked to me. The psychologist’s advice was very helpful to me. However, I’ve already hurt my husband. I had contemplated separation, but the counselor said to me, “Relax. You can help your husband. Your husband has an addiction disease, this disease is not worse than cancer.” Now I understand that the counselor was right. If I had divorced my husband, the situation would have exacerbated for our child, me, and him. Now, I can control myself.”*(p9)

### 3.2 Expectations

#### 3.2.1 Firm and Strict Rules and Regulations to Prevent Drug Smuggling

Most of the women objected the increasing availability of natural or synthetic drugs, which has raised the prevalence of drug abuse in the society. It is regrettable that addiction has now afflicted people from all walks of life and academic backgrounds. The wives of drug addicts in our study expected absolute restriction and proper control on drug smuggling in tandem with a definite and regulated plan for drug addiction prevention.

*“We want the head of the police and army to be sensitive about drug addiction. We expect the major players in drug trafficking to be brought to control. Addiction should be wiped out from the world.”* (p3)

#### 3.2.2 Expectation of Support from Family, Government, and Community

The spouses felt that they were the only ones who experienced and had to carry the burden, suffering and hardship because there was very little support from other sources such as family, community, and government. They wanted their voices to be heard by family, state, and society. In other words, they wanted to be understood. Ultimately, the women preferred not to depend on others because there is no proper encouraging rule and regulation to support them. Indeed, currently there are rules to support widows, but there are no rules to protect the wives of addicted men.

*“I want somebody to understand me, to listen to me and value me. I am lost and feel lonely; I need help and support. “* (p18)

*“If families come together; someone to help financially, someone to create a job opportunity, someone to offer moral support, someone to be friendly to my husband and to encourage him to participate in social activities, he will certainly improve.”* (p7)

*“I have two kids. If I had left my husband and children, I would have been comfortable. But what would have happened to my children? They would have become worse than their father or would’ve gotten hurt. They are innocent. They are the children of this society. Everyone is responsible. I will ask you a question: If something goes wrong, does it mean that everything should go wrong? Then, what about the future of this society and country? So, I will stay with my kids and protect them in every aspect of life (education, health, marriage…) provided that the government supports and understands me. Do I expect too much? Here I might benefit emotionally, but we would all benefit from a healthy community and society. Am I wrong?”*(p8)

## 4. Discussion

The present paper reports the findings of the first study in which needs and expectations of spouses of addicted men in Iran were interviewed in depth and qualitative methods were used.

Social problems are undoubtedly the complications that directly or indirectly affect all people, families, communities and states around the world. One of these social illnesses is drug addiction. Drugs are harmful not only due to its addiction effect but also because of its negative consequences on other family members. Addicts may die at young age; drug addicts become irresponsible; their behavior change; they talk of hallucinatory bliss and feel that they get peace of mind from drugs and may even talk about “the wonderful trip” (Ayub, 2013). Nevertheless, none of these descriptions is acceptable to those living with the addicted person (Barnard, 2005). Although addiction may have different impacts on different parts of the world based on their rule and regulation, globalization of the issue, (United Nations Office on Drugs and Crime, 1995; Levine, 2009) at different walks of life means that everyone including scholars, scientists, researcher, professionals, and state officials, should try to find a logical solution to solve addiction and its negative consequences on other family members (Orford, Templeton, Copello, Velleman, & Ibanga, 2010a).

The result of this study showed that the Iranian wives of addicted men are suffering severely from the negative impact and consequences of addiction. The UK, Italian researcher reported a similar finding related to family members (Orford, Velleman, Copello, Templeton, & Ibanga, 2010b).

It is essential that the needs and expectations of the family members of addicts be given due attention. This attention will help to achieve the goal of improving not only the patient treatment and care (National Treatment Agency for Substance Misuse, 2008), but also quality of life (Laudet, Morgen, & White, 2006). Women are integral part of the family life (MacKian, 2008). The first step in considering their needs and expectations is to take their husband’s treatment into account. With the treatment and recovery of the addicted husband, the whole phenomenon can be reversed. Treatment of addicted people will improve the individual’s welfare as well as the community’s health. To achieve this important goal, we should try to strengthen incentives, at first reduce or stop drug misuse, encourage addicts to pursue or continue treatment, engage them with exercise and sports, make health care services with high quality available, provide low cost or free treatment like offering them health care insurance coverage, and furnishing them with the combinations of therapies. A holistic approach, which is familycentered and community-based, can also help the addicts’ families to prevent relapse by addicted men, this finding was similar to the research results reported from UK (UK Drug Policy Commission, 2009; Macdonald et al., 2002; National Treatment Agency for Substance Misuse, 2008). The study of Copello et al. (2005) showed, using the family-focused interventions by health care provider need to be developed. Theses interventions can be broadly grouped into three types: (1) working with family members to promote the entry and engagement of substance misusers into treatment; (2) joint involvement of family members and substance misusing relatives in the treatment of the latter; and (3) responding to the needs of the family members in their own right.

Instrumental and financial short falls of the spouses is important. Instrumental and financial support must be considered by family or relatives, government, and supportive services. Unfortunately, lack of support system for the women living with addicted men in Iran renders the situation more complicated. To avoid the misuse of cash support, some limitations should be considered and other alternatives such as the supply of food packages containing essential nutrients such as protein products, dairy products, fruit, and vegetables should be provided for the families of addicted men who need to prevent the incidence of malnutrition. Other provisions such as clothing and a limited cash supply should also be offered in order to mitigate the financial burden, especially during follow-up of treatment. Dedicated, low-interest, self-employment loans as well as well-formulated supporting laws for these vulnerable and low-income classes of the community will reduce the burden.

The results showed that the spouses suffered from a sense of insecurity because of their husband’s unemployment or job instability and lack of a shelter of their own. Every family should have a job and a house although the first-degree relatives such as parents and in-laws are a source of help to these women to prevent the immediate breakdown of the family in the face of the husbands’ unemployment and high rents charges. Therefore, a calculative government’s support is of vital importance. If supportive systems do not pay heed to this problem, in addition to addiction, family breakdown and its consequences are likely to ensue. The UK drug policy commission members have some suggestion that we can use to improve the situation. These suggestions are: 1). the level and quality of direct support to help families in their own right need to be improved, 2). the stigma associated with drug dependency needs to be challenged; 3). the drug treatment system needs to be made more supportive and inclusive for families, 4). Leadership-responsibility for driving forward an agenda to enhance support for families needs to be placed with an identified champion at national and local levels, and 5). Information/knowledge development is essential for ensuring the adequacy and appropriateness of service provision-currently even the most basic data are lacking (UK Drug Policy Commission, 2009).

Addicted husbands were once the breadwinners of their respective families. These men require a sense of security to be able to resume a normal life. If the government provides job opportunities for these men, they will be able to meet the costs of daily life. The shelter problem can be solved if the authorities pay attention to the fact that this type of the family is unable to pay the heavy initial costs of public housing. There should be an alternative of low rate installments to enable this group of individuals to have and maintain a shelter. Another solution is the helping hand of donors and benefactors to the state welfare to intervene and solve the problems of these families. All these efforts could improve the quality of life in this family and can definitely help to reduce the risk of addiction in the society especially the next generation and will reduce the spread of crime and anti-social activities, There have been similar reports from Mexico regarding drug misuse causing family financial problems, thereby exacerbating existing family poverty (Orford et al., 1998, 2010b; Rahgozar et al., 2012).

The wives should not be blamed for their self-confessed misguided strategies which they adopted vis-à-vis their addicted husbands and other family members during the crisis. The women themselves clearly stated that they required spiritual, instrumental and financial support along with educational and training programs, so that they could effectively deal with their addicted husbands and family. There have been similar reports from UK regarding lack of information causing family don’t know what to do? (Macdonald et al., 2002; Orford, Copello, Velleman, & Templeton, 2010c).

One of the key requirements of the wives of addicted men at the time of admittance to the clinic is emotional and informational support. The professional should give the chance to the spouses to talk, chat, cry, and relieve themselves to reduce the stress, strain and get prepared better for educational programs such as how to deal with the husband’s addiction, how to achieve therapeutic success, tranquility, and improve the quality of life. The use of professional expertise in the health care system as well as the use of media and dedicated networks to provide education in the field of addiction as well as having provision of telephonic counseling agent could help to meet the informational needs (Orford, 2011).

The subjects underscored the action of the government to adopt a tough line on drug trafficking. The Iranian government has officially banned the cultivation and sales of narcotics, be that as it may. Still, much to surprise, drugs are readily available across the country because we share a long border with Afghanistan, which is one of the world’s largest producers of such substances. The smugglers somehow find their way to smuggle. The wives of addicted men; however, should take note that externalizing their husband’s addiction should not turn their attention away from internal factors (Parvizy, 2005).

The findings of this study also showed that the spouses felt that they were alone in their struggle against their husband’s addiction; (Orford et al., 2010b). Indeed, addiction is a complex phenomenon and its consequences can not solve single-handedly. There is no doubt that the families, health care system, community, state and government are needed to work together so as to create a drug-free country. Ultimately, in order to achieve a healthy society, everyone should make an effort to resolve the problem of addiction (UK drug policy commission, 2009).

This study suggests that the spouses had different emotional, informational, instrumental and financial needs, but the government and supportive organizations are not structural. Governmental and non-governmental organizations (NGOs) should consider providing them with required resources. The nurses in Iran do not pay attention to this vulnerable population; hence, nurses and family nurse practitioners should consider the spouses emotional and informational needs and increase women’s knowledge and facilitate easy access to information, and hold training sessions. Moreover, motivational counseling may be a critical intervention towards increasing motivation and social support for reducing their pain. The 5-Step Method has been developed in UK, based on the stress-strain-coping support model of addiction and the family. It is a pragmatic and flexible approach that considers the impact of addiction upon family members to be a very stressful experience that can be improved by working systematically with family members, through a series of steps covering the key components of the model; Listening nonjudgementally, providing relevant information, Discuss ways of responding to their needs and exploring ways of coping, Discussing social support and exploring sources of support, Arranging and establishing further help if needed (Copello, Templeton, Orford, & Velleman, 2010).

The present study assessed the needs and expectations of spouses of addicted men with various personal and socioeconomic characteristics. The impact of addiction on Iranian family, the social support interventions and assess their effects, may further promote the community health. Our participants were selected from Tehran, the capital of Iran. It is probable that women from other parts of Iran share different experiences. So, additional researches within other cities with different socio-cultural background will help us provide a mass of data related to the needs and expectations of spouses or other family members of addicts. Also, this inquiry is from the Iranian spouses of addicted men perspective. Women in other areas may have different views, or may have similar experiences. Therefore, further research are needed to enhance our understanding of the needs and expectations of these women in other countries to reach to a global conclusion and finding a concrete rule and regulation to help these women. The conclusions drawn point to the fact that current service provision, in treating the problem drug user in isolation, fails to address the needs of drug-affected families, and misses the opportunity to develop family-oriented support and treatment.

## 5. Conclusion

With this study, the researchers conclude that the needs and expectations of the families of addicts should be assessed on a case-by-case basis: individual by individual, family by family, and community by community till we reach to a concrete rule and regulation globally. We should handle the matter carefully by looking at different angles. Care should be taken to avoid the misuse of law by opportunists. In addition to the efforts to treat individuals afflicted with addiction, it is vital that the family members of addicts receive support and education. It is necessary to change the perspective of health care systems from patient-centered programs to family-centered and community-based ones.

## References

[ref1] American American Society of Addiction Medicine (2012). Definition of addiction.

[ref2] Angres D. H., Bettinardi-Angres K. (2008). The disease of addiction: Origins, treatment, and recovery. Disease A Month.

[ref3] Ayub Kabir (2013). Drug addiction: A social problem. Pamir Times.

[ref4] Barnard M. (2005). Drugs in the family: The impact on parents and siblings: Joseph Rowntree Foundation. http://www.jrf.org.uk/publications/drugs-family-impact-parents-andsiblings.

[ref5] Barnard M. (2007). Drug Addiction and Family.

[ref6] Copello A. G., Copello A. G., Velleman R. D., Templeton L. J. (2005). Family interventions in the treatment of alcohol and drug problems. Drug and alcohol review.

[ref7] Copello A. G., Templeton L., Velleman R. (2006). Family interventions for drug and alcohol misuse: Is there a best practice?. Current opinion in psychiatry.

[ref8] Copello A., Templeton L., Orford J., Velleman R. (2010). The 5-Step method: Principles and practice. Drugs: education, prevention and policy.

[ref9] Darrodi H., Bahrami F., Bahari F. (2010). Hope-Oriented Mental Rehabilitation and enhancement of marital satisfaction among couples with addicted husband. Iranian Rehabilitation Journal.

[ref10] Hsieh H. F., Shannon S. E. (2005). Three approaches to qualitative content analysis. Qualitative health research.

[ref11] Laudet A. B., Morgen K., White W. L. (2006). The role of social supports, spirituality, religiousness, life meaning and affiliation with 12-step fellowships in quality of life satisfaction among individuals in recovery from alcohol and drug problems. Alcoholism Treatment Quarterly.

[ref12] Levine H. G. (2009). Review of “The Globalization of Addiction: A Study in Poverty of the Spirit” by Bruce K. Alexander. Harm Reduction Journal.

[ref13] Macdonald D., Russell P., Bland N., Morrison A., De La Cruz C. (2002). Supporting families and carers of drug users: A review: Effective Interventions Unit.

[ref14] MacKian S. C. (2008). What the papers say: Reading therapeutic landscapes of women's health and empowerment in Uganda. Health & Place.

[ref15] Mirlashari J., Demirkol A., Salsali M., Rafiey H., Jahanbani J. (2012). Early childhood experiences, parenting and the process of drug dependency among young people in Tehran, Iran. Drug and alcohol review.

[ref16] Mokri A. (2002). Brief overview of the status of drug abuse in Iran. Arch Iranian Med.

[ref17] National Institute on Drug Abuse (2008). Addiction Research: A national imperative recomendations for the presidential transition. Provided by friends of NIDA.

[ref18] National Treatment Agency for Substance Misuse (2008). Supporting and involving carers: A guide for commissioners and providers.

[ref19] Nouri R. (2011). A comparison of women with addicted husbands and women with non-addicted husbands in terms of personality, characteristics, emotional feelings, and expressed satisfaction. Iranian Journal of Social Problems.

[ref20] Orford J. (2011). Re-Empowering Family Members Disempowered by Addiction: Support for Individual or Collective Action?. International Community Psychology, 163.

[ref21] Orford J., Copello A., Velleman R., Templeton L. (2010c). Family members affected by a close relative's addiction: The stress-straincopingsupport model. Drugs: education, prevention and policy.

[ref22] Orford J., Natera G., Davies J., Nava A., Mora J., Rigby K., Velleman R. (2013). Stresses and strains for family members living with drinking or drug problems in England and Mexico. Salud Mental.

[ref23] Orford J., Templeton L., Copello A., Velleman R., Ibanga A. (2010a). Working with teams and organizations to help them involve family members. Drugs: education, prevention and policy.

[ref24] Orford J., Velleman R., Copello A., Templeton L., Ibanga A. (2010b). The experiences of affected family members: A summary of two decades of qualitative research. Drugs: education, prevention and policy.

[ref25] Parvizy S., Naseri F., Seyed Fatemi N., Ghasem Zadeh Kakroudi F. (2010). Social factors contributing in women health in Tehran city: A qualitative study. Iranian Journal Nursing research.

[ref26] Parvizy S., Nikbahkt A., Pournaghash Tehrani S., Shahrokhi S. (2005). Adolescents’perspectives on addiction: Qualitative study. Nursing & Health Sciences.

[ref27] Rahgozar H., Mohammadi A., Yousefi S., Piran P. (2012). The Impact of Father's Addiction on His Supportive and Economic Role in the Family and Social Relations and Socialization of the Family Members: The Case of Shiraz, Iran. Asian Social Science.

[ref28] Ray G, T, Mertens J, R., Weisner C. (2007). The excess medical cost and health problems of family members of persons diagnosed with alcohol or drug problems. Med Care Journal.

[ref29] Streubert Speziale H., Carpenter D. (2011). Qualitative research in nursing.

[ref30] Tafreshi Seyed Hesamoddin (2012). Statistics of drug addiction in Iran. Razi journal.

[ref31] UK Drug Policy Commission (2009a). Adult family members and careers of dependent drug users: prevalence, social cost, resource savings and treatment responses.

[ref32] UK Drug Policy Commission (2009b). Supporting the supporters: Families of drug misusers.

[ref33] United Nations Office on Drugs and Crime, Ctr, V. I., & Austria (2011). World Drug Report, 2011. No.: ISBN 978-92-1-148262-1, 272.

[ref34] United Nations Office on Drugs and Crime (1995). The social impact of drug abuse.

[ref35] Velleman R., Templeton L. (2003). Alcohol, drugs and the family: results from a long-running research programme within the UK. European Addiction Research Journal.

[ref36] Velleman R., Templeton L. (2007). Understanding and modifying the impact of parents’substance misuse on children. Advances in Psychiatric Treatment.

